# Effect of a Professional Information Campaign on the Use of Sevoflurane in Pediatric Anesthesia

**DOI:** 10.1111/pan.70095

**Published:** 2025-11-28

**Authors:** Lionel Bouvet, Aurélie Boutarin, Mathilde De Queiroz, Laurent Zieleskiewicz

**Affiliations:** ^1^ Hospices Civils de Lyon Hôpital Femme Mère Enfant, Université Lyon 1 Lyon France; ^2^ Assistance Publique Des Hôpitaux de Marseille Hôpital Nord, Aix Marseille University Marseille France

**Keywords:** carbon footprint, global warming, halogenated anesthetic, pediatric anesthesia, sustainable anesthesia

1

Climate change, due to the increase in greenhouse gas emissions linked to human activities, represents a major challenge for public health. Healthcare systems in developed countries contribute approximately 5%–10% of total carbon dioxide equivalent (CO_2_e) emissions [1]. Among these emissions, operating rooms are a major contributor mainly due to the use of halogenated anesthetic gases, such as sevoflurane. In fact, sevoflurane has a global warming potential over 100 years (GWP_100_) 130 times greater than carbon dioxide (CO_2_), though it is significantly less than desflurane [2]. Furthermore, halogenated anesthetics contribute to atmospheric pollution and introduce forever chemicals (PFAS) into aquatic ecosystems. Despite its environmental impact, sevoflurane is widely used in pediatric anesthesia because of its ease of administration, particularly when intravenous access is not available and in younger children. However, intravenous anesthetics such as propofol may be associated with fewer complications (e.g., laryngospasms, agitation, nausea, vomiting, and postoperative pain [3, 4, 5]) and have a considerably lower carbon footprint than halogenated anesthetics [2], although propofol presents some ecotoxic effects on aquatic life.

We recently reported that an information campaign led by sustainable anesthesia groups at the Hospices Civils de Lyon resulted in a significant reduction in the carbon footprint related to halogenated anesthetics in adults, primarily due to a 90% reduction in desflurane use, which was replaced by sevoflurane, without a significant increase in propofol use [6]. To address the specificities of pediatric anesthesia, we conducted an informational campaign proposing new protocols to promote the rational use of sevoflurane in pediatric anesthesia. We then aimed to assess the impact of this information campaign on the use of propofol and sevoflurane at the pediatric operating theater of our institution.

In November 2023, a questionnaire was distributed to anesthesiologists and nurse anesthetists working in the pediatric operating room of the Femme‐Mère‐Enfant Hospital, Lyon, France. This survey aimed to assess their knowledge of the ecological and medical issues related to the use of sevoflurane in pediatric anesthesia. Subsequently, an informational campaign was conducted in February 2024, consisting of three meetings with oral presentations and email dissemination of protocols suggesting the use of intravenous propofol rather than inhaled halogenated anesthesia for specific pediatric patient groups (Figure 1).

**FIGURE 1:**
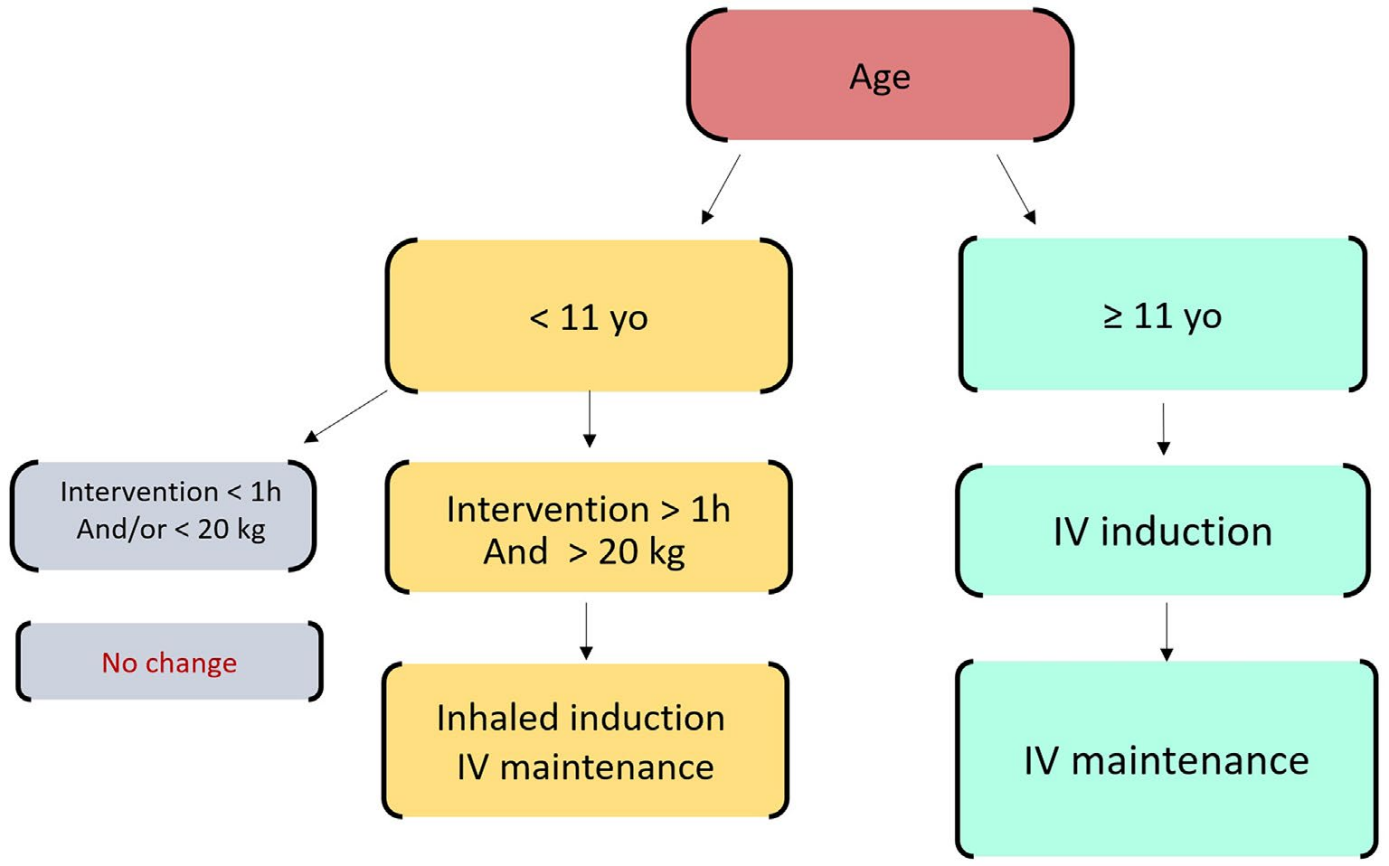
Sevoflurane‐sparing protocol.

A retrospective cohort study was then performed to compare medical data from pediatric patients operated on during two periods: September–October 2023 (pre‐intervention) and March–April 2024 (postintervention). We analyzed the hypnotic agents used for anesthesia induction and maintenance, as well as the incidence of anesthesia‐related complications. Data were expressed as medians (interquartile ranges) or numbers (percentages). Statistical analyses were conducted using Chi‐square and Mann–Whitney *U* tests, with a significance threshold of *p* < 0.05. Effect sizes were expressed as absolute differences in percentages or medians, with corresponding 95% confidence intervals. For continuous variables, 95% confidence intervals of median differences were calculated using the Hodges–Lehmann estimator. The study was approved by the institutional review board (Comité d'Éthique des Hospices Civils de Lyon, N°23‐5326).

Out of 57 professionals surveyed, 40 (70%) responded to the questionnaire. Among respondents, 32 (80%) were already aware of the environmental impact of sevoflurane use.

A total of 1063 pediatric patients were analyzed, including 586 from the pre‐intervention period and 477 from the postintervention period. The median age of the patients was 11 years (8–14 years), with a median weight of 38 kg (26–53 kg). Most patients were male (*n* = 0.648; 61%), and most surgeries were scheduled procedures (*n* = 0.723; 69%), primarily orthopedic, digestive, and otolaryngological surgeries. Patient and surgery characteristics were comparable between the two periods, except for the median procedure duration, which was significantly longer in the pre‐intervention period (103 [76–153] min vs. 88 [65–134] min; median difference: −13 min; 95% confidence interval: −18 min to −8 min; *p* < 0.001).

The rate of intravenous induction with propofol increased significantly from 206/586 (35%; 95% confidence interval: 31% to 39%) general anesthesia in the pre‐intervention period to 210/477 (44%; 95% confidence interval: 40% to 49%) in the postintervention period (absolute increase of 9%; 95% confidence interval: 3% to 15%; *p* = 0.003), while intravenous maintenance increased from 41/586 (7%; 95% confidence interval: 5% to 10%) in the pre‐intervention period to 70/477 (15%; 95% confidence interval: 12% to 19%) in the postintervention period (absolute increase of 8%; 95% confidence interval: 4% to 11%; *p* = 0.032). A peripheral venous line was inserted before the induction of anesthesia in 144/586 (25%; 95% confidence interval: 22% to 29%) children during the first period, compared to 168/477 (35%; 95% confidence interval: 31% to 40%) children during the second period (absolute increase of 11%; 95% confidence interval: 5% to 16%; *p* = 0.0002). No significant differences were observed in the rates of anesthesia‐related complications between the two periods.

Transitioning to sustainable anesthesia may be challenging in pediatrics, where inhalational induction and nitrous oxide use remain common and difficult to replace, potentially making educational interventions alone insufficient to achieve large‐scale change without system‐level constraints and continuous feedback [7]. Despite the limitations related to the design of this preliminary study, our findings suggest that information campaigns can effectively raise awareness among healthcare professionals regarding the environmental impact of anesthetic practices and encourage behavior change to reduce environmental harm without compromising patient safety and comfort. Regarding the latter, this initiative was implemented in a department that prioritizes patient comfort, using nonpharmacological strategies to reduce perioperative anxiety [8], and, when indicated, pharmacological premedication. Local anesthesia is systematically applied before intravenous catheter placement. Moreover, the protocol may have encouraged greater shared decision‐making, enabling children to choose between intravenous propofol and inhalational induction, rather than defaulting to the latter. This aspect warrants further investigation. In parallel, efforts have been made to reduce fresh gas flow rates during inhalational anesthesia to limit sevoflurane consumption and emissions. Together, these initiatives represent a further step toward sustainable healthcare practices, combining high‐quality patient care with less environmental impact. Further research is needed to quantify CO_2_e reductions from changing pediatric anesthesia practices, assess their long‐term sustainability, and evaluate the environmental impact of increased propofol use.

## Funding

The authors have nothing to report.

## Conflicts of Interest

The authors declare no conflicts of interest.

## Data Availability

The data that support the findings of this study are available from the corresponding author upon reasonable request.
